# Identification of biomarkers co-associated with M1 macrophages, ferroptosis and cuproptosis in alcoholic hepatitis by bioinformatics and experimental verification

**DOI:** 10.3389/fimmu.2023.1146693

**Published:** 2023-04-06

**Authors:** Shasha Hou, Dan Wang, Xiaxia Yuan, Xiaohuan Yuan, Qi Yuan

**Affiliations:** ^1^Department of Life Science and Engineering, Jining University, Jining, China; ^2^College of Life Science, Mudanjiang Medical University, Mudanjiang, China

**Keywords:** alcoholic hepatitis, M1 macrophage, ferroptosis, cuproptosis, WGCNA

## Abstract

**Backgrounds:**

Alcoholic hepatitis (AH) is a major health problem worldwide. There is increasing evidence that immune cells, iron metabolism and copper metabolism play important roles in the development of AH. We aimed to explore biomarkers that are co-associated with M1 macrophages, ferroptosis and cuproptosis in AH patients.

**Methods:**

GSE28619 and GSE103580 datasets were integrated, CIBERSORT algorithm was used to analyze the infiltration of 22 types of immune cells and GSVA algorithm was used to calculate ferroptosis and cuproptosis scores. Using the “WGCNA” R package, we established a gene co-expression network and analyzed the correlation between M1 macrophages, ferroptosis and cuproptosis scores and module characteristic genes. Subsequently, candidate genes were screened by WGCNA and differential expression gene analysis. The LASSO-SVM analysis was used to identify biomarkers co-associated with M1 macrophages, ferroptosis and cuproptosis. Finally, we validated these potential biomarkers using GEO datasets (GSE155907, GSE142530 and GSE97234) and a mouse model of AH.

**Results:**

The infiltration level of M1 macrophages was significantly increased in AH patients. Ferroptosis and cuproptosis scores were also increased in AH patients. In addition, M1 macrophages, ferroptosis and cuproptosis were positively correlated with each other. Combining bioinformatics analysis with a mouse model of AH, we found that ALDOA, COL3A1, LUM, THBS2 and TIMP1 may be potential biomarkers co-associated with M1 macrophages, ferroptosis and cuproptosis in AH patients.

**Conclusion:**

We identified 5 potential biomarkers that are promising new targets for the treatment and diagnosis of AH patients.

## Introduction

Alcohol-associated liver disease (ALD) is a serious public health problem worldwide (1). Alcoholic hepatitis (AH) is one of the phenotypes of ALD, which is mainly caused by a long history of excessive alcohol consumption and a recent history of severe alcohol abuse (2). AH presents a clinical syndrome characterized by jaundice and liver injury. In the past 50 years, corticosteroids are still the main therapeutic drugs, and no effective new drugs have been successfully developed (3). Although corticosteroids increase short-term survival in AH patients, approximately 40% of patients do not respond to treatment (4, 5). In recent years, the rapid development of high-throughput sequencing technology has promoted the understanding of AH (6, 7). Therefore, it is urgently needed to identify new biomarkers in AH patients by bioinformatics analysis, which will facilitate the development of new treatment strategies.

The liver plays a major regulatory role in alcohol metabolism and immune monitoring. Hepatocytes exposed to alcohol cause damage and death due to oxidative stress, which in turn produces a variety of inflammatory factors to activate the inflammatory response and immune cells (8). Macrophages are important cells of innate immune system. The complex functional variability and adaptability of macrophages to different infection situations are based on their extensive phenotypic plasticity. naive macrophages (M0) can be polarized into classically activated macrophages (M1 macrophages) and alternately activated macrophages (M2 macrophages), which perform proinflammatory or anti-inflammatory functions, respectively (9). Previous studies have shown that damaged hepatocytes activate the NF-κB signaling pathway under alcohol metabolism, which releases a series of chemokines and inflammatory mediators, ultimately promoting macrophage M1 polarization (10, 11). In addition, Cho et al. revealed that G-CSF improved liver function by promoting macrophage M2 polarization in alcohol-fed mice (12).

Ferroptosis is a unique type of cell death regulation, which is caused by iron accumulation, excessive production of reactive oxygen species (ROS) and excessive lipid peroxidation (13). Cuproptosis is a recently discovered type of cell death caused by the direct binding of copper to the lipidized proteins of the mitochondrial tricarboxylic acid cycle (TCA) (14). Alcohol metabolism in hepatocytes affects mitochondrial function and produces a large number of ROS, leading to elevated lipid peroxidation. Thus, the progression of AH is closely related to ferroptosis and cuproptosis. As recently reported, intestinal sirtuin1 (SIRT1) deficiency protects mice from alcohol-induced inflammation by mitigating hepatic ferroptosis (15). Melatonin inhibits ferroptosis by activating Nrf2-ARE signaling pathway, thus alleviating alcohol-induced liver injury (16). Copper metabolism in the liver is still being explored. Cuproptosis regulates immune cell infiltration and is used to construct risk assessment models for hepatocellular carcinoma (HCC) (17, 18). However, the role of ferroptosis and cuproptosis in AH patients needs to be further explored.

In this study, we downloaded and integrated transcriptome data from AH patients. Potential biomarkers of AH patients were identified based on the M1 macrophages, ferroptosis and cuproptosis scores, and these biomarkers were validated using public datasets and a mouse model of AH. Finally, we identified 5 potential biomarkers: aldolase A (ALDOA), Collagen type III alpha 1 (COL3A1), lumican (LUM), thrombospondin-2 (THBS2) and tissue inhibitor of metalloproteinase-1 (TIMP1). These potential biomarkers could provide new targets for the diagnosis and treatment of AH patients.

## Materials and methods

### Data set download and evaluation

Gene expression data were downloaded from the Gene Expression Integrated Database (GEO)(http://www.ncbi.nlm.nih.gov/geo/) with accession numbers GSE28619 (19), GSE103580 (20), GSE155907 (21), GSE142530 (7) and GSE97234 (22), the basic information of our selected samples is shown in **Supplementary Table S1**. The batch effect between GSE28619 and GSE103580 was corrected using the “sva” R package (23). The intersected genes between GSE28619 and GSE103580 were obtained *via* online Venn Diagram analysis (jvenn, http://jvenn.toulouse.inra.fr/app/index.html).

### Analysis of immune cells

The CIBERSORT algorithm was used to calculate the proportion of 22 types of immune cells with normalized gene expression data (24). Correlations between immune cells were evaluated using the “corrplot” R package. Based on the characteristics of immune cells, principal component analysis (PCA) was to cluster the normal liver samples and AH samples. Specifically, the “stats” R package was used for PCA analysis. Firstly, z-score was performed on the expression profile, and then prcomp function was used for dimension reduction analysis to obtain the matrix after dimension reduction.

### Gene set variation analysis

The “GSVA” R package (25) was used to calculate the scores of ferroptosis gene set and cuproptosis gene set. A total of 64 ferroptosis-related genes were obtained from MigDB (**Supplementary Table S2**). The 16 cuproptosis-related genes were collected from previous literature (26) (**Supplementary Table S3**).

### Weighted gene co-expression network analysis

WGCNA is an algorithm for constructing gene clustering modules based on similar gene expression patterns. We used the “WGCNA” R package (27) to construct a co-expression network of genes from normal liver samples and AH samples. The concrete steps are as follows: First, the optimal soft-thresholding power was calculated and selected. Second, the adjacency matrix was constructed based on the selected soft-thresholding power and transformed into a topological overlap matrix. Third, hierarchical clustering tree was established to cluster high-coexpression genes into the same module. Finally, M1 macrophages, ferroptosis and cuproptosis scores were used as characteristics to calculate the correlation between module genes and traits. In this study, we screened hub genes based on threshold weight > 0.2, and Cytoscape software (version 3.9.1) was used to visualize the gene networks.

### Functional enrichment analysis

To further clarify biological functions and signaling pathways of candidate genes, we used the “clusterProfiler” R package (28) for functional enrichment analysis, including gene ontology (GO) and Kyoto Encyclopedia of Genes and Genomes (KEGG) analysis. The result of functional enrichment analysis was visualized using the “GOplot” R package (29).

### Analysis of differentially expressed genes

We used the “limma” R package (30) to calculate differentially expressed genes (DEGs) between normal liver samples and AH samples. DEGs were obtained by threshold standard |log2(FC)| > 1, p-value < 0.05. The volcano and heatmap plots were visualized *via* the “ggplot2” and “pheatmap” R packages.

### Machine learning

By intersecting DEGs and WGCNA hub genes, 27 candidate genes associated with AH patients were identified. For these 27 candidate genes, two machine-learning techniques were used to further screen potential genes in AH patients. The least absolute shrinkage and selection operator (LASSO) is an algorithm used for regularization to improve prediction accuracy and model comprehensibility, and to select variables. We utilized the LASSO algorithm to screen potential biomarkers in AH patients by “glmnet” R package (31). Support vector machines (SVM) is a powerful method whose goal is to establish a threshold between two classes that allows label prediction based on single or multiple feature vectors. We used SVM method to screen potential biomarkers in AH patients by “kernlab” R package (32). The intersection of the results between the two methods were obtained *via* online Venn Diagram analysis (jvenn, http://jvenn.toulouse.inra.fr/app/index.html). To further assess the ability of biomarkers to distinguish AH samples from normal liver samples, we performed receiver operating characteristic (ROC) analysis using the “pROC” R package (33).

### A mouse model of AH

As previously mentioned, a mouse model of chronic alcohol plus single binge drinking was established (34). The alcoholic diet was purchased from TROPHIC (Nantong, China). Ten male mice aged 6-8 weeks were fed a liquid control diet for 5 days, then mice were randomly divided into two groups (ethanol-fed group and pair-fed group, n = 5 per group). The ethanol-fed group was fed a Lieber DeCarli liquid diet containing 5% ethanol for 10 days. Then mice were given a single dose of 20% ethanol (5g/kg body weight) by gavage. The pair-fed group was fed with ethanol-free, isocaloric control liquid diet for 10 days. Then mice were given a single dose of dextrin maltose (5g/kg body weight) by gavage. Euthanasia was performed 9 hours after gavage. All animal experiments were performed with the approval of the Experimental Animal Ethics Committee of Mudanjiang Medical University.

### Blood biochemical assays

Blood samples of mice were centrifuged at 1000×g for 10 min to obtain serum. Serum alanine aminotransferase (ALT) and aspartate aminotransferase (AST) levels were detected by kits of Nanjing Jiancheng Bioengineering Institute (Nanjing, China).

### Content analysis of malondialdehyde (MDA) and glutathione

Liver tissues were homogenized according to the instructions, and MDA and GSH levels were detected by kits of Nanjing Jiancheng Bioengineering Institute (Nanjing, China).

### Histology and immunofluorescence

Paraffin or cryostat sections were prepared as described previously (35). Paraffin sections were stained with hematoxylin and eosin (H&E). For fluorescence double staining, cryostat sections were incubated with anti-iNOS antibody (Santa Cruz Biotechnology, Santa Cruz, CA, USA) and anti-F4/80 antibodies (BioLegend, San Diego, CA, USA), followed by incubation with Alexa Fluor 488- or 594-conjugated secondary antibodies (Jackson ImmunoResearch, West Grove, PA, USA). Sections were evaluated under a microscope (DP71, OLYMPUS) of both bright-field and fluorescence microscopy (200 × magnification).

### Real-time quantitative PCR

Total RNA was isolated from liver tissues using TRIzol reagent (TransGen Biotech, Beijing, China), and cDNAs were synthesized using FastKing RT Kit (TIANGEN, Beijing, China). RT-qPCR analysis was performed using SuperReal PreMix Plus (TIANGEN, Beijing, China). The primer sequences were listed in **Supplementary Table S4**. Data were analyzed using the 2^-ΔΔCT^ method and normalized to β-actin (*Actb*) expression.

### Western blot

Total protein from liver tissue was extracted using RIPA lysis buffer (Solarbio, Beijing, China) containing protease inhibitor cocktail (MedChemExpress, Princeton, NJ, USA). The samples were incubated at 99°C for 5 min and separated at 115 V by SDS-PAGE for 1 h. The proteins were transferred to PVDF membranes and incubated at 200 mA for 1 h. The membrane was plugged with 5% milk powder for 1 hour and incubated overnight at 4°C with the following primary antibody: anti-FDX1 (Absin, Shanghai, China), anti-GPX4, anti-ACSL4, anti-SLC31A1 and anti-β-actin (Affinity, Cincinnati, OH, USA). HRP-conjugated goat anti-rabbit IgG was used as secondary antibodies. All bands were quantified with an automated digitizing system (ImageJ).

### Statistical analysis

All data were presented as mean ± SD and analyzed using GraphPad Prism (version 8.3.0) and R (version 4.2.1). Significant differences in animal experiments were determined by three independent experiments. Differences of continuous variables between two groups were compared using Student’s t-test analysis. *P*<0.05 was considered statistically significant.

## Results

### Overview of study design

**Figure 1:**
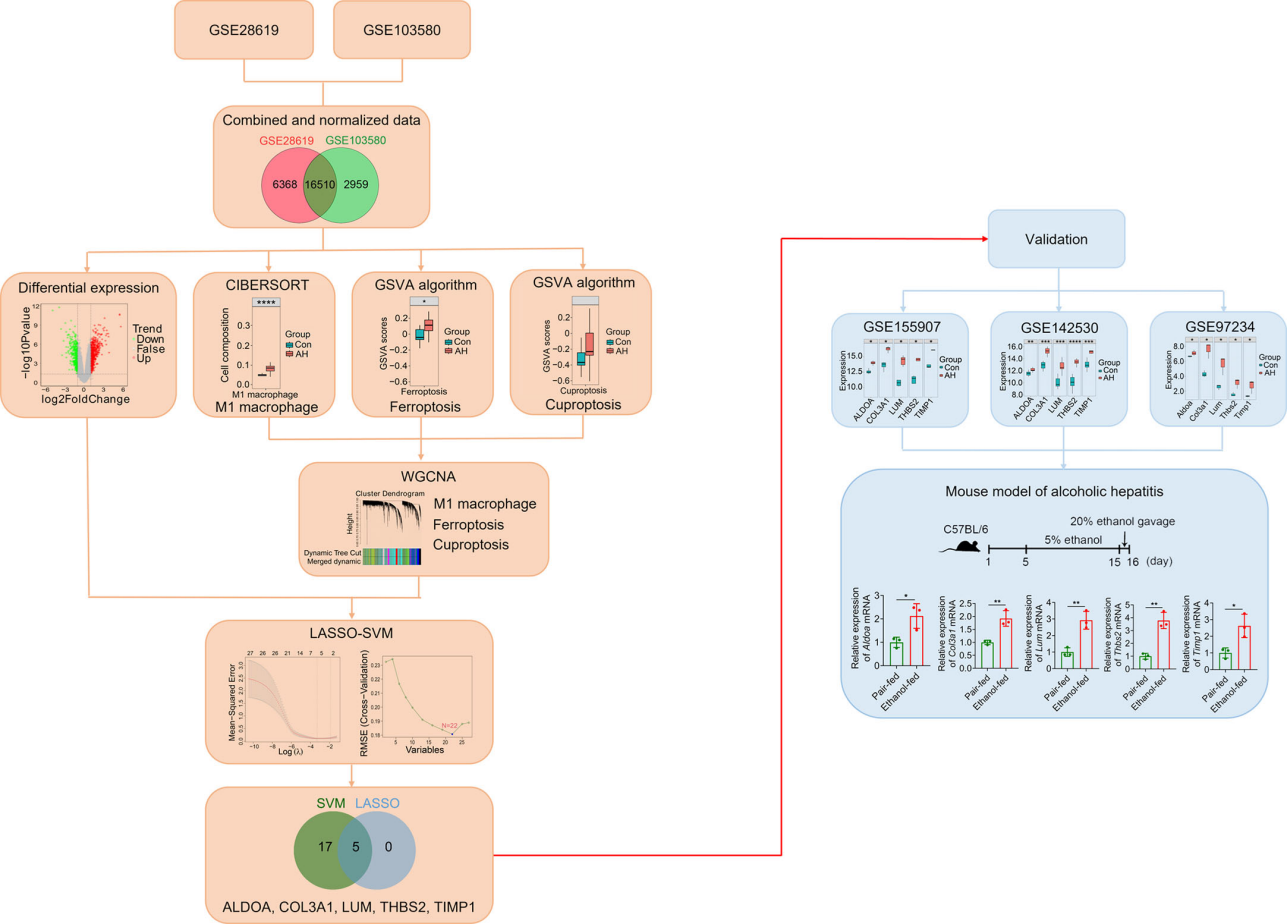
Overall schematic diagram of the study design. *p < 0.05, **p < 0.01, ***p < 0.001, ****p < 0.0001.

The overall design scheme of our current study is shown in **Figure 1**. First, we combined and normalized data of GSE28619 and GSE103580. Secondly, CIBERSORT method was used to analyze immune cells, GSVA algorithm was used to calculate scores of ferroptosis and cuproptosis, and WGCNA was used to screen hub genes related to M1 macrophages, ferroptosis and cuproptosis. Third, we analyzed DEGs using the “limma” package and intersected DEGs with hub genes. Fourth, we identified biomarkers of AH based on LASSO-SVM algorithm. Finally, we used GEO data (GSE155907, GSE142530 and GSE97234) and a mouse model of AH to validate potential biomarkers.

### Normalization of dataset

Both GSE28619 and GSE103580 datasets are chip data. The GSE28619 dataset was based on the GPL570 platform (Affymetrix Human Genome U133 Plus 2.0 Array) and included 7 normal liver samples and 15 AH samples. The GSE103580 dataset was based on the GPL13667 platform (Affymetrix Human Genome U219 Array) from which 13 AH samples were selected. The two datasets were merged and batch removed. The results before and after normalization are shown in **Supplementary Figure S1A**. As shown in the Venn Diagram (**Supplementary Figure S1B**), 22,878 and 19,469 probes were identified in GSE28619 and GSE103580, respectively, and 16,510 intersected genes were selected from two datasets for subsequent bioinformatics analysis.

### Analysis of immune infiltration in normal liver and AH samples

**Figure 2:**
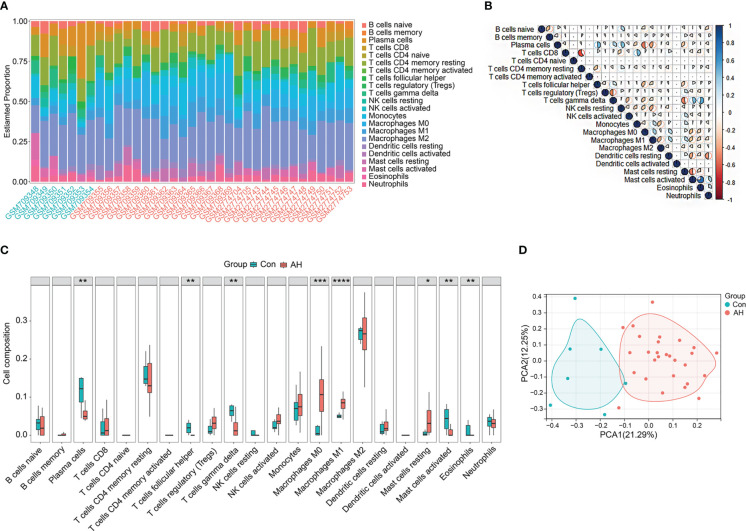
Immune infiltration in control group and AH group. **(A)** Bar charts of 22 types of immune cells in selected samples. Green name is normal liver sample, red name is AH sample. **(B)** Correlation heatmap of 22 types of immune cells, blue is positive correlation, red is negative correlation, color intensity represents the degree of correlation. **(C)** Boxplot of difference analysis of immune cells between control group (Con) and AH group (AH). **(D)** Scatter plot of PCA results. *p < 0.05, **p < 0.01, ***p < 0.001, ****p < 0.0001.

CIBERSORT, the deconvolution algorithm reported by Newman et al., characterizes cell composition in complex tissues based on normalized gene expression profiles (24). Based on this algorithm, we calculated the infiltration of 22 types of immune cells in normal liver samples and AH samples. The bar chart shows the abundance of different immune cell subsets in each sample (**Figure 2A**). We further analyzed the correlation between 22 immune cell subsets. As shown in the correlation heatmap (**Figure 2B**), activated mast cells showed the most significant positive correlation with eosinophils (r = 0.75), while CD8 T cells showed the most significant negative correlation with CD4 memory resting T cells (r = -0.61). Next, we analyzed the difference in immune cells between normal liver samples (control group) and AH samples (AH group). Compared with control group, M0 macrophages, M1 macrophages and resting mast cells were significantly increased in AH group, while plasma cells, helper follicular T cells, gamma delta T cells, activated mast cells and eosinophils were significantly decreased in AH group (**Figure 2C**). PCA analysis of control group and AH group and performed based on 22 types of immune cells. As shown in **Figure 2D**, AH group was completely separated from control group, suggesting that activation of immune cells could be a significant feature of AH patients.

### Co-expression modules of M1 macrophages, ferroptosis and cuproptosis in AH patients

**Figure 3:**
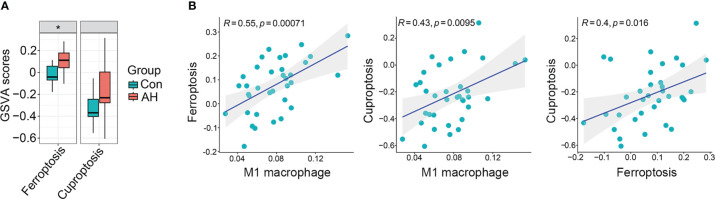
M1 macrophages, ferroptosis and cuproptosis were positively correlated with each other. **(A)** Boxplot of difference analysis of ferroptosis and cuproptosis GSVA scores between control group (Con) and AH group (AH). **(B)** Correlation analysis of M1 macrophage, ferroptosis and cuproptosis. *p < 0.05.

**Figure 4:**
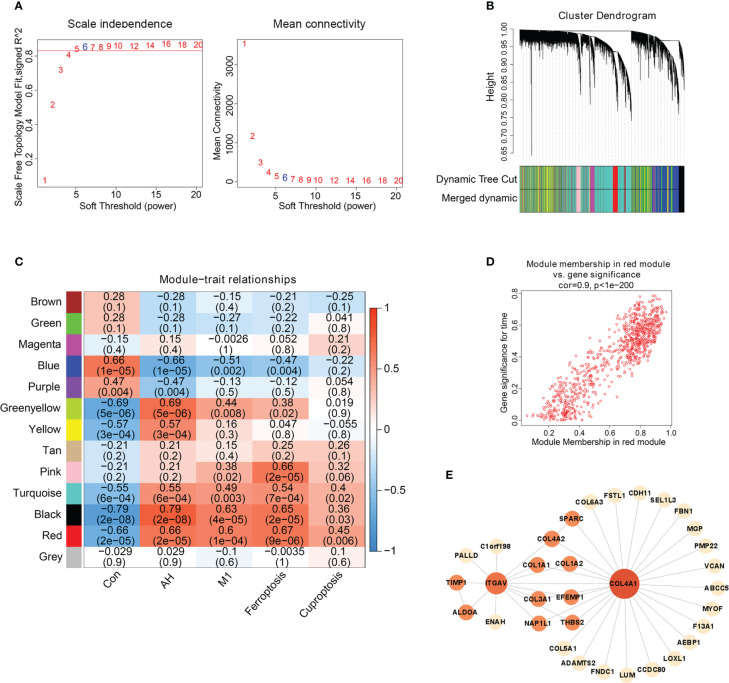
Hub gene screening based on WGCNA. **(A)** Scale-free fitting index analysis of soft-thresholding powers. **(B)** Cluster dendrogram. **(C)** Module-trait correlation heatmap, red is positive correlation, blue is negative correlation. **(D)** Scatter plot of red module. Horizontal axis (MM) represented the correlation between genes and modules, and vertical axis (GS) represented the absolute value of correlation between genes and phenotypic characteristics. **(E)** The network of hub genes.

We found that M1 macrophages were the most differentiated immune cells between control and AH groups (**Figure 2C**). At the same time, the GSVA algorithm was used to calculate scores of ferroptosis and cuproptosis. Compared with the control group, ferroptosis scores of the AH group were significantly increased. Although there was no statistical difference in GSVA scores of cuproptosis, there was a increasing trend (**Figure 3A**). In addition, correlation analysis showed that M1 macrophages, ferroptosis and cuproptosis were positively correlated with each other (**Figure 3B**). To further explore the role of genes co-associated with M1 macrophages, ferroptosis and cuproptosis in AH patients, we used CIBERSORT’s M1 macrophage results, ferroptosis and cuproptosis scores as characteristic data for WGCNA analysis. The power value is set as β value when the correlation coefficient between connectivity K and logarithm logarithm (P(k)) reaches 0.83. A scale-free topological network (β = 6) was established (**Figure 4A**). Based on selected soft-thresholding power, a hierarchical clustering tree was established to cluster high-coexpression genes into same module and color code them (**Figure 4B**). Next, Spearman correlation analysis was used to draw module-trait relationship heatmap for 13 transcription modules identified and evaluate relationship between modules (**Figure 4C**). We found that red module was closely correlated with M1 macrophages, ferroptosis and cuproptosis, and was also highly correlated with AH traits. Therefore, this module was identified as hub module (**Figures 4C, D**). The module contains 834 genes, including 33 genes and 41 edges with threshold weight > 0.2 (**Figure 4E**). These genes are considered as hub genes.

### Candidate genes co-associated with M1 macrophages, ferroptosis and cuproptosis in AH patients

**Figure 5:**
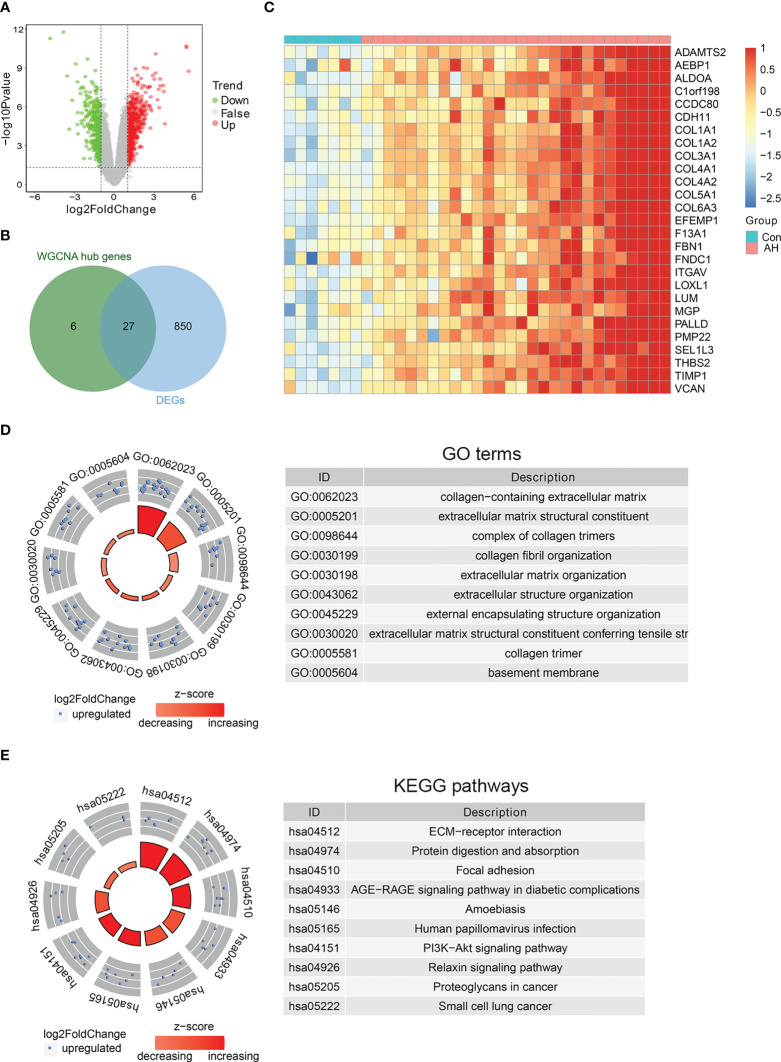
Identification of biomarkers co-associated with M1 macrophages, ferroptosis and cuproptosis. **(A)** Volcano plot of DEGs between control and AH groups. **(B)** Venn diagram of intersection genes between DEGs and hub genes. **(C)** The heatmap of 27 candidate genes. **(D)** GO enrichment analysis. **(E)** KEGG enrichment analysis.

To further identify biomarkers associated with AH patients, DEGs analysis was performed on gene expression data from control and AH groups. There were a total of 877 DEGs, including 519 up-regulated genes and 358 down-regulated genes (**Figure 5A**) (**Supplementary Table S5**). The intersection of DEGs with hub genes related to M1 macrophages, ferroptosis and cuproptosis was performed to obtain 27 candidate genes (**Figure 5B**). The heatmap shows expression of these candidate genes in each sample (**Figure 5C**). Functional enrichment analysis was conducted for the above 27 candidate genes. The top 10 significantly enriched GO terms and KEGG pathways are shown separately in **Figures 5D, E** (see **Supplementary Table S6** for details). AGE-RAGE and PI3K-AKT signaling pathway are associated with inflammation and oxidative stress. This suggests that M1 macrophages, ferroptosis and cuproptosis may be related to each other through the above signaling pathways. In addition, the results of functional enrichment analysis showed that the common high expression of M1 macrophages, ferroptosis and cuproptosis may activate extracellular matrix (ECM)-related signaling pathways.

### Identification of biomarkers co-associated with M1 macrophages, ferroptosis and cuproptosis *via* machine learning

**Figure 6:**
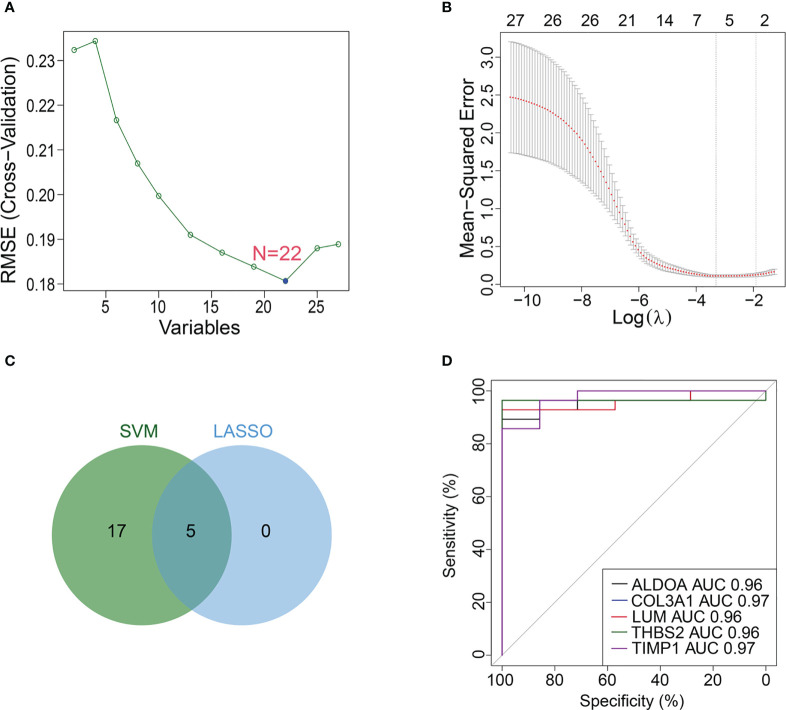
Machine learning identifies potential biomarkers. **(A, B)** Biomarkers were analyzed using LASSO regression and SVM algorithm. **(C)** Venn diagram of overlapping biomarkers between LASSO regression and SVM algorithm. **(D)** ROC curve of 5 potential biomarkers.

**Figure 7:**
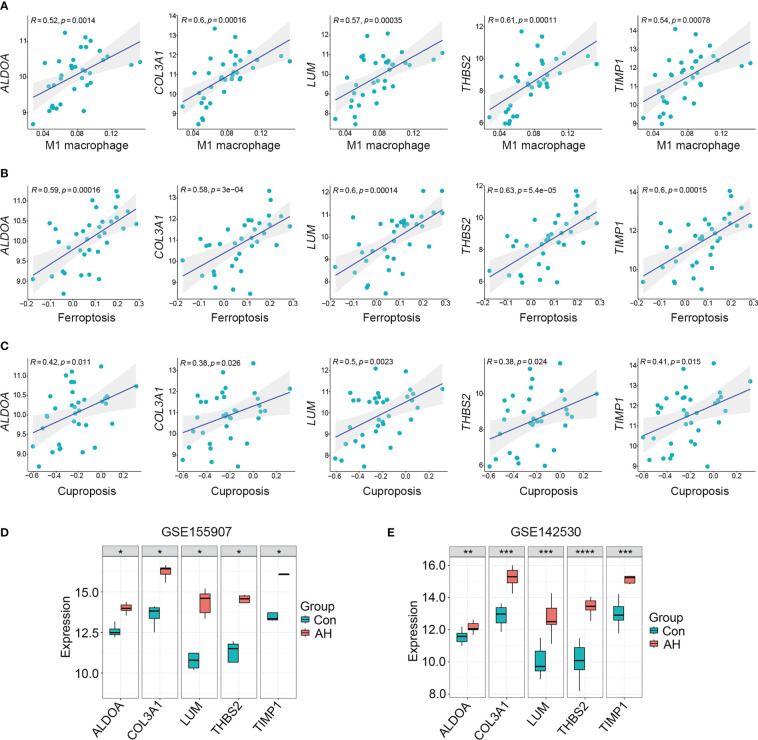
5 potential biomarkers were positively correlated with M1 macrophage, ferroptosis and cuproptosis. **(A–C)** Correlation analysis of 5 potential biomarkers with M1 macrophage, ferroptosis and cuproptosis. **(D)** Expression levels of 5 potential biomarkers in GSE155907 dataset. **(E)** Expression levels of 5 potential biomarkers in GSE142530 dataset. *p < 0.05, **p < 0.01, ***p < 0.001, ****p < 0.0001.

For the above 27 candidate genes, SVM and LASSO regression algorithms were used to screen potential biomarkers co-associated with M1 macrophages, ferroptosis and cuproptosis. According to the results of ten fold cross-validation in SVM algorithm, 22 feature genes were identified (**Figure 6A**, **Table 1**). The coefficients of LASSO versus log (λ) are shown in **Figure 6B** that 5 feature genes were obtained (**Table 1** and **Supplementary Table S7**). Finally, 5 genes selected by two machine learning algorithms were overlapped, including ALDOA, COL3A1, LUM, THBS2 and TIMP1 (**Figure 6C**). To assess predictive accuracy of these biomarkers, ROC curves of 5 genes were analyzed (**Figure 6D**). The AUC values indicated that 5 biomarkers co-associated with M1 macrophages, ferroptosis and cuproptosis had excellent diagnostic values. Next, we analyzed the correlation between 5 potential biomarkers and M1 macrophage, ferroptosis and cuproptosis. The analysis results showed that 5 potential biomarkers were positively correlated with M1 macrophage, ferroptosis and cuproptosis (**Figures 7A–C**), and 5 potential biomarkers were also positively correlated with each other (**Supplementary Figure S2**). In addition, we validated 5 potential biomarkers using GSE155907 and GSE142530 datasets. In two validation datasets, consistent with training dataset, all 5 genes in AH group were up-regulated, with statistical significance (**Figures 7D, E**). Combined with the above results, 5 genes co-associated with M1 macrophages, ferroptosis and cuproptosis can be used as potential biomarkers in AH patients.

**Table 1:** Feature genes obtained by machine learning.

Machine learning	Feature genes
SVM	CCDC80, COL5A1, ITGAV, CDH11, VCAN, THBS2, COL4A2, COL4A1, SEL1L3, PMP22, C1orf198, FBN1, PALLD, COL1A2, COL3A1, EFEMP1, TIMP1, ALDOA, LUM, ADAMTS2, COL6A3, COL1A1
LASSO	ALDOA, COL3A1, LUM, THBS2, TIMP1

### Potential biomarkers were validated in a mouse model of AH

**Figure 8:**
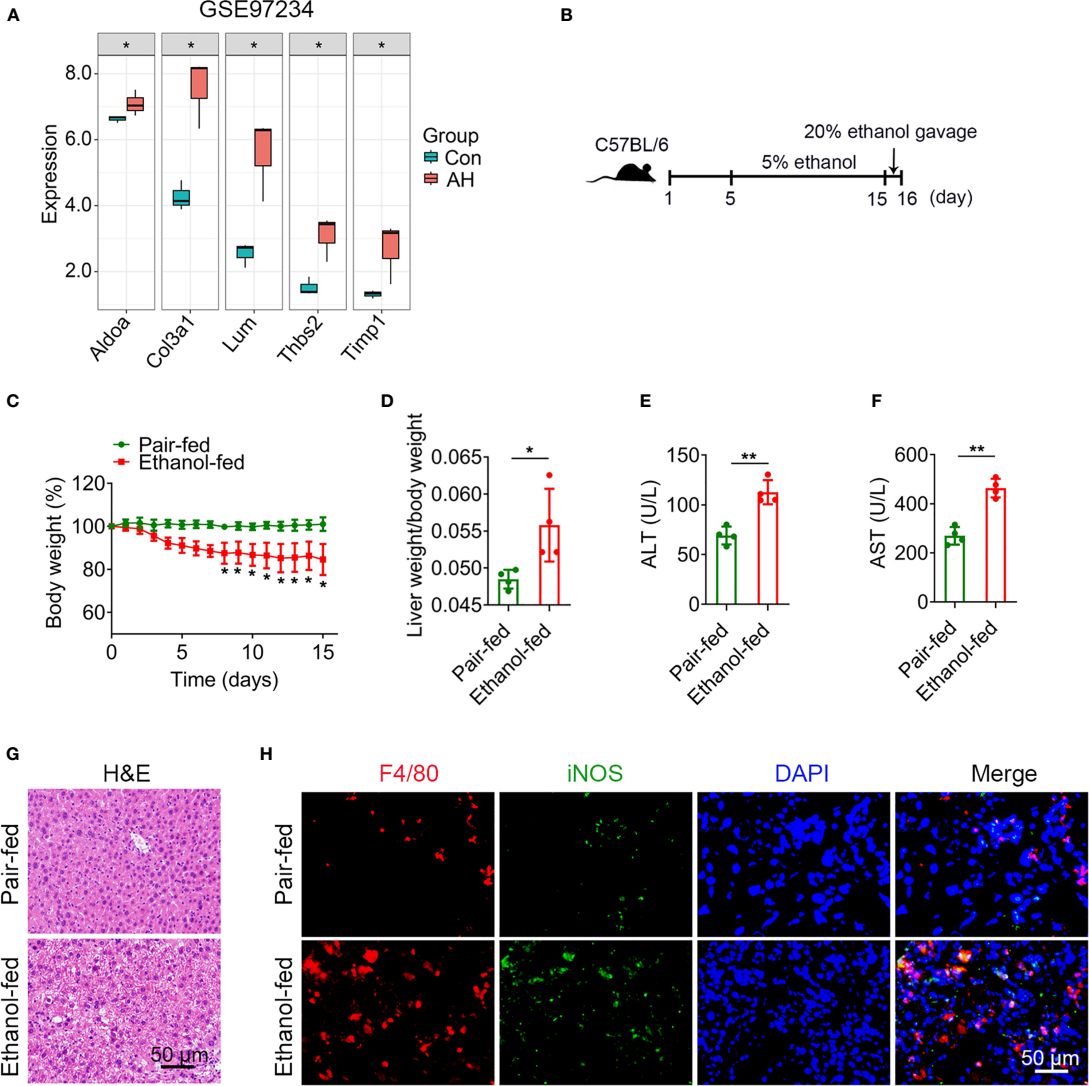
Alcohol exposure promotes infiltration of M1 macrophages. **(A)** Expression levels of 5 potential biomarkers in GSE97234 dataset. **(B–H)** Groups of C57BL/6 mice (n = 5 per group) were fed ethanol liquid diet or ethanol free control liquid diet. **(B)** Schematic diagram of a chronic binge eating model. **(C)** Changes in body weight. **(D)** Liver weight/body weight change ratio. **(E, F)** Serum ALT and AST levels. **(G)** Representative H&E staining of liver tissues. Scale bar, 50 μm. **(H)** Double immunofluorescence staining of F4/80 and iNOS in liver tissues. Nuclei were stained with DAPI. Scale bar, 50 μm. *P < 0.05, **P < 0.01.

To verify 5 potential biomarkers, we first analyzed mouse dataset (GSE97234) and found that the expression levels of 5 genes were significantly up-regulated in AH group (**Figure 8A**). Based on the above analysis, we conducted experimental verification in mice. We established a chronic plus single binge alcohol model, which has been widely used to study the pathogenesis of AH (**Figure 8B**). Compared with pair-fed mice, ethanol-fed mice had a significant decrease in body weight (**Figure 8C**) and a significant increase in liver weight/body weight ratio (mean, 0.056 vs. 0.048, *P* = 0.028) (**Figure 8D**). ALT and AST, important indicators of liver injury, were significantly elevated in ethanol-fed mice than in pair-fed mice (ALT, mean, 112.7 vs. 69.1, *P* = 0.001) (AST, mean, 464.0 vs. 269.7, *P* = 0.0003) (**Figures 8E, F**). H&E staining showed that ethanol feeding resulted in necrosis of hepatocytes and morphological changes of liver tissues, suggesting more severe liver injury in ethanol-fed mice than in pair-fed mice (**Figure 8G**).

**Figure 9:**
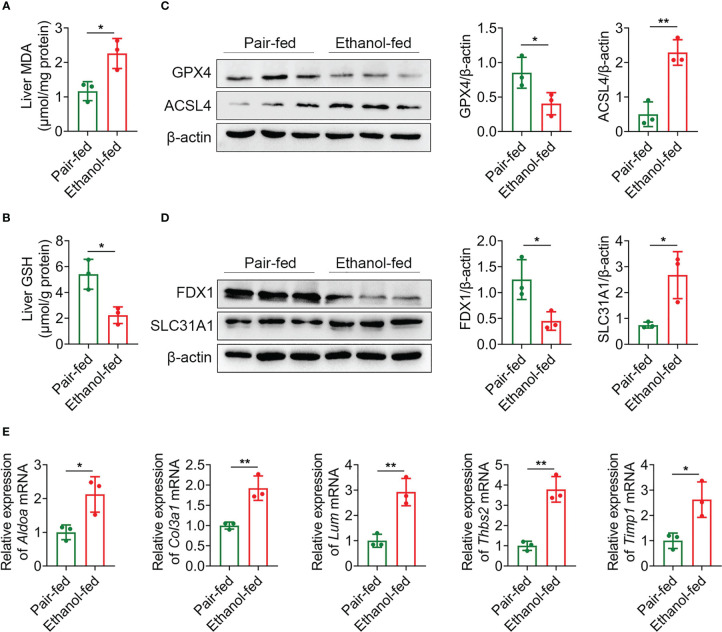
Alcohol exposure promotes the expression of ferroptosis, cuproptosis and potential biomarkers. **(A–E)** Groups of C57BL/6 mice (n = 5 per group) were fed ethanol liquid diet or ethanol free control liquid diet. **(A)** Content of MDA in liver tissues. **(B)** Content of GSH in liver tissues. **(C)** The protein expression levels of GPX4 and ACSL4 in liver tissues were determined using western blot analysis. The densities of protein were quantified using densitometry. GPX4 and ACSL4 were normalized to β-actin. **(D)** The protein expression levels of FDX1 and SLC31A1 in liver tissues were determined using western blot analysis. The densities of protein were quantified using densitometry. FDX1 and SLC31A1 were normalized to β-actin. **(E)** The mRNA levels of *Aldoa*, *Col3a1*, *Lum*, *Thbs2* and *Timp1* in liver tissues were measured using RT-qPCR analysis. The results were normalized to *Actb*. *P < 0.05, **P < 0.01.

Macrophages play a crucial role in regulating liver homeostasis and hepatic injury. By double immunofluorescence staining of liver tissues with F4/80 (a marker of macrophage) and iNOS (a marker of M1 macrophage), we found that M1 macrophages (F4/80 and iNOS double positive cells) were almost not expressed in pair-fed mice, while the expression of M1 macrophage marker protein was increased in ethanol-fed mice, suggesting the infiltration of more M1 macrophages (**Figure 8H**). Lipid peroxide and GSH are crucial markers of ferroptosis. MDA is considered to be the end product of the lipid peroxidation process. The MDA and GSH levels of liver tissues were detected by kits. Compared with pair-fed mice, MDA levels (mean, 2.26 vs. 1.17, *P* = 0.021) were significantly increased and GSH levels (mean, 2.24 vs. 5.41, *P* = 0.014) were significantly decreased in ethanol-fed mice (**Figures 9A, B**). In addition, the well-identified markers of ferroptosis, GPX4 and ACSL4, were detected in liver tissues by western blot. As shown in **Figure 9C**, the protein expression levels of GPX4 (mean, 0.40 vs. 0.85, *P* = 0.048) were significantly down-regulated and the protein expression levels of ACSL4 (mean, 2.29 vs. 0.50, *P* = 0.004) were significantly up-regulated in ethanol-fed mice compared with pair-fed mice. Known biomarkers of cuproptosis, including FDX1 and SLC31A1 were determined. We also detected the expression levels of FDX1 and SLC31A1 in liver tissues by western blot. the protein expression levels of FDX1 (mean, 0.45 vs. 1.25, *P* = 0.031) were significantly down-regulated and the protein expression levels of SLC31A1 (mean, 2.68 vs. 0.75, *P* = 0.022) were significantly up-regulated in ethanol-fed mice compared with pair-fed mice (**Figure 9D**). Combined with the above results, alcohol consumption significantly promoted the infiltration of M1 macrophages, the expression of ferroptosis and cuproptosis.

The expression levels of *Aldoa*, *Col3a1*, *Lum*, *Thbs2* and *Timp1* mRNA were detected by RT-qPCR. As shown in **Figure 9E**, compared with pair-fed mice, the expression levels of *Aldoa* (mean, 2.12 vs. 1.00, *P* = 0.027), *Col3a1* (mean, 1.92 vs. 1.00, *P* = 0.007), *Lum* (mean, 2.92 vs. 1.00, *P* = 0.005), *Thbs2* (mean, 3.79 vs. 1.00, *P* = 0.002) and *Timp1* (mean, 2.62 vs. 1.00, *P* = 0.021) in liver tissues of ethanol-fed mice were significantly increased. These experimental results further support that 5 genes as potential biomarkers for AH patients.

## Discussion

In this study, biomarkers co-associated with M1 macrophages, ferroptosis and cuproptosis were identified in AH patients. The CIBERSORT algorithm was used to calculate infiltration of 22 types of immune cells. Ferroptosis and cuproptosis scores were calculated using GSVA algorithm. By WGCNA and LASSO-SVM analysis, we found that ALDOA, COL3A1, LUM, THBS2 and TIMP1 were potential biomarkers in AH patients. These biomarkers were validated in GEO datasets and a mouse model of AH.

Excessive alcohol consumption directly damages hepatocytes, which in turn induces immune cell infiltration and secretion of inflammatory factors, ultimately leading to overactivation of inflammatory cascade (36, 37). For example, neutrophil infiltration and high expression of pro-inflammatory factors (TNF-α and IL-1β) promote the progression of alcohol-related inflammatory response (38). In addition, chronic alcohol consumption leads to upregulation of M1 macrophage-related markers (39). In this study, we used the CIBERSORT algorithm to assess the difference in immune cells between normal liver and AH samples. We found that M1 macrophages were significantly increased in AH patients. These findings provide new insights into immune cell infiltration in AH patients based on transcriptomic analysis.

Clinical calculators such as the Model of End-stage Liver Disease (MELD) score can predict patient mortality and guide clinical treatment strategies (40). However, MELD score is not specifically designed to predict AH. Clinically, biomarkers for AH prediction have not been identified. Therefore, the discovery of new biomarkers for AH prediction is an urgent area of research. In recent years, with the development of high-throughput sequencing technology, the identification of disease-related biomarkers based on transcriptomic analysis has been widely studied. In previous studies, a prognostic model of hepatocellular carcinoma was established using WGCNA analysis of macrophage-related genes (41). The prognostic model based on ferroptosis and epithelial-mesenchymal transition state helps predict overall survival of hepatocellular carcinoma (42). Cuproptosis-related subtypes predict tumor microenvironments and drug candidates in hepatocellular carcinoma (43). However, biomarkers for AH prediction still need further analysis. In this study, we used WGCAN and LASSO-SVM analysis to identify 5 AH biomarkers co-associated with M1 macrophages, ferroptosis and cuproptosis in AH patients.

ALDOA is a key metabolic enzyme in glycolysis pathway. High expression of ALDOA is associated with poor prognosis in hepatocellular carcinoma (44). COL3A1 is a fibrous collagen found in connective tissue. Previous studies have shown that COL3A1 is involved in the progression of liver fibrosis (45). The levels of type III collagen formation and degradation were significantly increased in ALD patients compared to healthy individuals (46). In addition, LUM has been identified as a biomarker for advanced fibrosis in non-alcoholic fatty liver disease (47). THBS2 is a novel biomarker for predicting the prognosis of metastatic pancreatic ductal adenocarcinoma (48). Manzardo et al. analyzed miRNA expression in alcoholics to further characterize the genetic influence of alcoholism and the influence of alcohol consumption on predicted target mRNA expression, which involved THBS2 (49). In mice treated with ethanol and CCl_4_, down-regulation of TIMP1 effectively inhibited hepatic fibrosis and activation of hepatic stellate cell (50). Clinical studies have found that alcohol consumption in adolescents leads to elevated serum TIMP1 concentrations (51). We first reported that ALDOA, COL3A1, LUM, THBS2 and TIMP1 were highly expressed in AH and associated with M1 macrophages, ferroptosis and cuproptosis. Further studies with larger clinical cohorts and basic studies are needed to confirm these biomarkers.

## Conclusion

In summary, we used CIBERSORT algorithm to analyze 22 types of immune cells, and M1 macrophages were the most significantly increased immune cells in AH patients. By combining bioinformatics analysis with a mouse model of AH, we identified 5 potential biomarkers co-associated with M1 macrophages, ferroptosis and cuproptosis. Further study of these biomarkers can provide new ideas and basis for understanding the disease progression and targeted therapy of AH patients.

## Data availability statement

The original contributions presented in the study are included in the article/**Supplementary Material**. Further inquiries can be directed to the corresponding author.

## Ethics statement

The animal study was reviewed and approved by The Experimental Animal Ethics Committee of Mudanjiang Medical University.

## Author contributions

Conception and design: QY. Data collection and analysis: SH, DW and QY. Manuscript writing: SH and XXY. Manuscript revising: XHY. All authors contributed to the article and approved the submitted version.
