# Supplementary material for: Identification of biomarkers co-associated with M1 macrophages, ferroptosis and cuproptosis in alcoholic hepatitis by bioinformatics and experimental verification
Source: Front Immunol. 2023 Apr 6;14:1146693. doi: 10.3389/fimmu.2023.1146693 (PMC10117880; doi:10.3389/fimmu.2023.1146693)
Supplement: Supplementary file 1 [file DataSheet_1.pdf]

Supplementary Material

Identification of biomarkers co-associated with M1 macrophages, ferroptosis and cuproptosis in alcoholic hepatitis by bioinformatics and experimental verification

Shasha Hou[1], Dan Wang[2], Xiaxia Yuan[1], Xiaohuan Yuan[2], Qi Yuan[2*]

[1] Department of Life Science and Engineering, Jining University, Jining, P.R. China.

[2] College of Life Science, Mudanjiang Medical University, Mudanjiang, P.R. China.

*** Correspondence:**
Qi Yuan, College of Life Science, Mudanjiang Medical University, No.3 Tongxiang Road, Mudanjiang, 157011, P.R. China. Tel/Fax: 86-0453-6984401; E-mail: yuanqi@mdjmu.edu.cn

Supplementary Figures

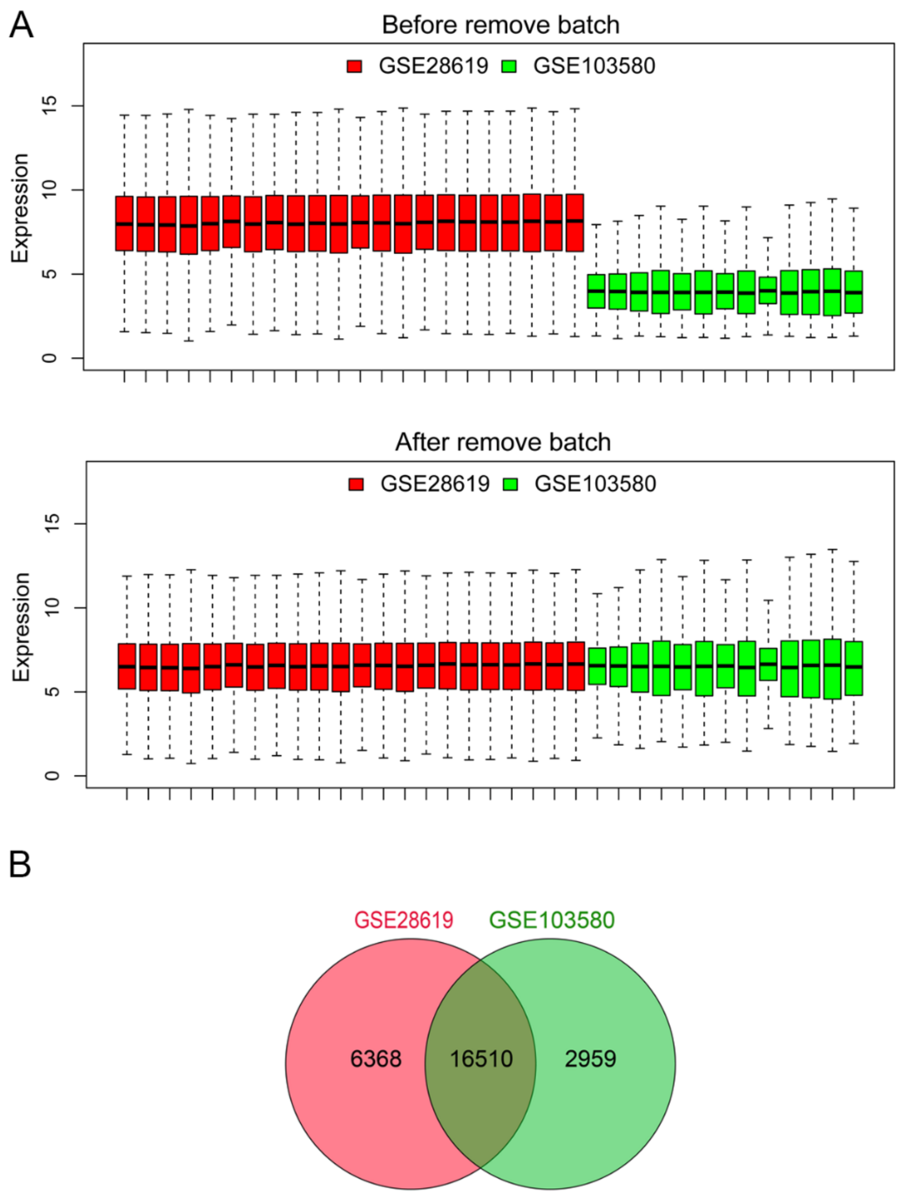

Supplementary Figure S1.
Data Preparation.
(A) Boxplots of expression genes before and after normalization of GSE28619 and GSE103580. (B) Venn diagram of GSE28619 and GSE103580 expression genes.

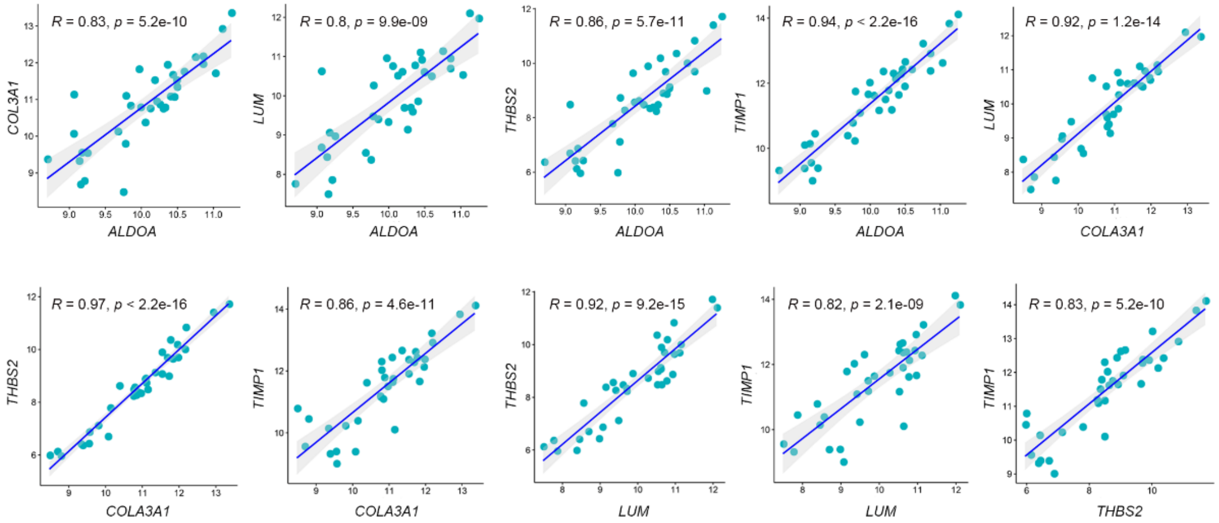

Supplementary Figure S2
5 potential biomarkers were positively correlated with each other.
Correlation analysis of 5 potential biomarkers.
