# Supplementary material for: Identification of biomarkers co-associated with M1 macrophages, ferroptosis and cuproptosis in alcoholic hepatitis by bioinformatics and experimental verification
Source: Front Immunol. 2023 Apr 6;14:1146693. doi: 10.3389/fimmu.2023.1146693 (PMC10117880; doi:10.3389/fimmu.2023.1146693)
Supplement: Supplementary file 2 [file Table_1.docx]

**Table S1. The basic information of our selected samples**

| Datasets | Normal liver samples | AH samples |
| --- | --- | --- |
| GSE28619  (Homo sapiens) | GSM709348, GSM709349, GSM709350, GSM709351, GSM709352, GSM709353, GSM709354 | GSM709355, GSM709356, GSM709357, GSM709358, GSM709359, GSM709360, GSM709361, GSM709362, GSM709363, GSM709364, GSM709365, GSM709366, GSM709367, GSM709368, GSM709369 |
| GSE103580  (Homo sapiens) |  | GSM2774704, GSM2774705, GSM2774743, GSM2774744, GSM2774745, GSM2774746, GSM2774747, GSM2774748, GSM2774749, GSM2774750, GSM2774751, GSM2774752, GSM2774753 |
| GSE155907  (Homo sapiens) | GSM4715485, GSM4715486, GSM4715487, GSM4715488 | GSM4715489, GSM4715490,  GSM4715491, GSM4715492,  GSM4715493 |
| GSE142530  (Homo sapiens) | GSM4231384, GSM4231385,  GSM4231386, GSM4231387,  GSM4231388, GSM4231389,  GSM4231390, GSM4231391,  GSM4231392, GSM4231393,  GSM4231394, GSM4231395 | GSM4231368, GSM4231369  GSM4231370, GSM4231371  GSM4231372, GSM4231373, GSM4231374, GSM4231375  GSM4231376, GSM4231377 |
| GSE97234  (Mus musculus) | GSM2559987, GSM2559988,  GSM2559989, GSM2559990 | GSM2559994, GSM2559995,  GSM2559996 |
