# Supplementary material for: Identification of biomarkers co-associated with M1 macrophages, ferroptosis and cuproptosis in alcoholic hepatitis by bioinformatics and experimental verification
Source: Front Immunol. 2023 Apr 6;14:1146693. doi: 10.3389/fimmu.2023.1146693 (PMC10117880; doi:10.3389/fimmu.2023.1146693)
Supplement: Supplementary file 5 [file Table_4.docx]

**Table S4. Primer sequences used for RT-qPCR**

| **Gene** | **Species** | **Primer sequence (5'–3')** |
| --- | --- | --- |
| *Aldoa* | Mouse | F: CACGAGACACTGTACCAGAAGG |
|  |  | R: TTGTCTCGCCATTGGTTCCTGC |
| *Col3a1* | Mouse | F: GACCAAAAGGTGATGCTGGACAG |
|  |  | R: CAAGACCTCGTGCTCCAGTTAG |
| *Lum* | Mouse | F: TATACCAACAGTTAATGAAAATCTTGA |
|  |  | R: GGATTGCCATCCAAGCGCAGAT |
| *Thbs2* | Mouse | F: GTATGGAGGGAAGGACTGTGTC |
|  |  | R: ACTTGGCTCCAGGAAAACACGG |
| *Timp1* | Mouse | F: TCTTGGTTCCCTGGCGTACTCT |
|  |  | R: GTGAGTGTCACTCTCCAGTTTGC |
| *Actb* | Mouse | F: CATTGCTGACAGGATGCAGAAGG |
|  |  | R: TGCTGGAAGGTGGACAGTGAGG |
